# Interrater Agreement Between Paramedic CTAS Scores and an Electronic CTAS Tool: A Retrospective Study Across Eastern Ontario

**DOI:** 10.1155/emmi/3209833

**Published:** 2026-09-07

**Authors:** Michael Slatter, Pria Nippak, Nadia Nandlall, Housne Begum, Julie Sinclair, Thula Kandasamy, Ben De Mendonca

**Affiliations:** ^1^ School of Health Services Management, Ted Rogers School of Management, Toronto Metropolitan University, Toronto, Canada, ryerson.ca; ^2^ The Regional Paramedic Program for Eastern Ontario (RPPEO), Ottawa, Canada; ^3^ Ottawa Paramedic Service, Ottawa, Canada

**Keywords:** Canadian hospital emergency departments (CEDs), electronic smartphone application, paramedics, patient safety risks, the Canadian triage and acuity scale (CTAS)

## Abstract

**Objectives:**

To assess interrater agreement between the Canadian triage and acuity scale (CTAS) scores assigned by paramedics and those assigned by a paramedic rater using an electronic smartphone application, aiming to identify training gaps and potential patient safety risks associated with CTAS scoring in Canadian hospital emergency departments (CEDs).

**Methods:**

A retrospective analysis was conducted using 192,326 CTAS‐level call data collected from electronic patient care records of nine paramedic services. Data were entered into an electronic CTAS smartphone app. The calls were categorized based on contact level and the sample size. Minitab software and VassarStats were used to calculate the quadratic‐weighted Cohen’s kappa (*k*) coefficient.

**Results:**

A retrospective analysis of 1651 paramedic call reports from across Eastern Ontario compared paramedic‐assigned CTAS scores with those generated using an eCTAS smartphone tool. Overall agreement between raters was substantial (*k* = 0.78), with 55.24% (95% CI: 52.8, 57.66) agreement for CTAS on contact and 52.88% (95% CI: 50.44, 55.31) for CTAS on departure (*k* = 0.76). Paramedics assigned more low‐acuity CTAS 4–5 scores than the eCTAS tool on both contact (39% vs. 31%) and departure (40% vs. 31%). Additionally, 11%–12% of CTAS 2 calls were downgraded by paramedics to CTAS 4 or 5, compared with 7% when eCTAS was used. Cohen’s kappa demonstrated near‐perfect agreement for CTAS 1 scores, fair agreement for CTAS 2 and 3, slight agreement for CTAS 4, and moderate agreement for CTAS 5 categories. Complaint‐specific analyses showed the greatest disagreement in the gastrointestinal and substance overdose/misuse categories.

**Conclusions:**

This study found near‐perfect agreement between paramedic documentation and the paramedic smartphone application for CTAS‐1 in acute patients, with moderate agreement for other levels. Further studies should include multiple raters to better understand the implications of using a smartphone application.

## 1. Introduction

The Canadian Triage and Acuity Scale (CTAS) was initially developed and utilized within Canadian hospital emergency departments to assist in the sorting of patients based on their acuity of illness or injury. Paramedics in Ontario began using the CTAS system to create a standardized nomenclature between paramedics, ambulance communications officers (ACOs), and the ED staff to assess patient acuity [1]. Initial system‐wide CTAS training occurred in the early 2000s, with a refresher in 2017 when the latest version of the CTAS standard was implemented in 2016 [2, 3]. Subsequently, CTAS training became a core part of the Ontario Paramedic Community College curriculum, and students are expected to have the knowledge to implement CTAS upon entering the workforce. Paramedics are required to assign up to three CTAS levels for all patients they encounter. A CTAS level is assigned for CTAS on contact with the patient, CTAS on departure from the scene, and CTAS on arrival at an ED [1]. CTAS is determined through a series of gathered information that includes the patient’s age, the presenting complaint, and a quick first impression, followed by first‐ and second‐order modifiers that result in a generated CTAS level from 1 to 5 [4]. A minimum of two CTAS levels must be assigned, and this is initially completed through memory, as CTAS booklets or smartphone applications are not conducive to use in the current environment in which paramedics work in. With limited refresher training opportunities due to current work, budget, and system constraints, there may be some variability in the accuracy of CTAS assignments. In addition to CTAS retention issues among paramedics, CTAS remains a subjective, fluid system that changes over time in relation to treatments or interventions, despite the fact that CTAS is meant to create a common language for healthcare providers [5]. Accordingly, it is extremely important to compare the same datasets against an expert or a validated electronic CTAS tool to ensure that agreement on CTAS assignments is preserved. Common methods used have included simulated scenarios presented either through a computer program or on a paper‐based presentation [6–9]. To ensure baseline knowledge of CTAS levels, all of the studies that have looked at the rater’s ability to assign an appropriate CTAS level have provided training as part of the study, or the participants had a minimum of 5 years of experience conducting triage assessments prior to taking part in any study [6]. These procedural similarities between studies highlight the need for healthcare professionals to be up to date with their knowledge of CTAS levels. Studies have also demonstrated that the experience of the healthcare professional appears to be another relevant factor impacting CTAS assignment accuracy, with experienced nurses performing better than those with less experience [8].

More recently, studies have examined the reliability and accuracy of CTAS scores between healthcare professionals and computer programs or applications, specifically paramedics [7] or triage nurses [6, 8] versus a computer program. To assess interrater reliability in CTAS assignments across studies, researchers typically used quantitative data analysis approaches including both linear‐weighted and quadratic weighted kappa (*k*) statistics [10]. The inclusion criteria for these studies ensured that participants were employed in a component of the healthcare field, mainly nursing, and were clinically active. Patient samples in both simulated and clinical environments included patients aged greater than 17 years.

Across all study designs, consensus between raters was observed independent of the types of participants [7, 11–13]. The other common finding was that there tends to be a risk of undertriage for patients who fall into a riskier category, meaning that CTAS 2 and 3 levels were labeled 3 and 4, respectively [14]. However, overtriage has also been reported to potentially have an impact on healthcare resources [15]. To date, most studies have focused on comparing various clinical professional scores between nurses and nurses or paramedics and nurses, or nurses to that of an electronic CTAS tool, but no studies have explored the reliability between paramedics and an electronic CTAS tool, such as a smartphone application. There are studies that show that paramedics can use the CTAS guidelines successfully with experience and fresh training [9]. However, there is a lack of information on how well paramedics assign CTAS in the field, given the variability in CTAS training and memory performance that exists across healthcare professionals working in the field [16].

Further variability arises with the growing number of alternative programs and applications used by paramedics. For example, the Province of Ontario recently announced that Base Hospitals can create programs to take patients to alternate destinations, either to non‐ED healthcare settings or to use resources like taxis for transport to a healthcare destination.

More recently, CTAS has been used for additional purposes beyond assessment. For example, paramedic services have been using CTAS to allow for patient priority system (PPS) considerations that include bypassing the closest hospital with CTAS 3, 4, or 5 patients, suggesting that CTAS assignment may become even more important in the provision of care by emergency services. [9]. Within Canada, the Province of Ontario is also moving to a new dispatching system called the Medical Priority Dispatch System or MPDS. MPDS relies on paramedics providing only a single CTAS on departure level to the ACO, rather than the standard three: CTAS on contact, CTAS on departure, and CTAS on arrival at the ED. This increased reliance on CTAS to plan and direct healthcare services strongly supports a re‐examination of CTAS assignment by paramedics. The aim of this study was to determine if interrater agreement exists between documented paramedic‐assigned CTAS scores in the field against a paramedic rater using an electronic CTAS smartphone application. Also, the goal of the study was to explore paramedics’ use of CTAS in the field to generate a hypothesis about potential gaps in training or implications for patient safety.

## 2. Materials and Methods

### 2.1. Study Design

This study was a retrospective analysis of the CTAS levels documented on electronic call reports submitted for the fiscal year from April 1, 2021, to March 31, 2022, to the Regional Paramedic Program for Eastern Ontario (RPPEO). The annual call volume was 192,326. Using the determined sample size for each CTAS level, the first subsequent reports were selected for review once sorted. This ensured a random sample from each of the nine paramedic services under the purview of the RPPEO. The data collected included CTAS on contact, CTAS on departure, chief complaint, primary problem, primary problem code, return priority, incident history, final problem, vital signs, and patient assessment. These data were used to populate an electronic CTAS smartphone application, which generated a CTAS level that was examined for agreement with the paramedic’s documented CTAS level. The smartphone CTAS tool used was validated by the CTAS National Working Group of Canada, the Canadian Association of Emergency Physicians, and the National Emergency Nurses Association to provide healthcare professionals with appropriate CTAS training supports for both their educational advancement and clinical triage decision‐making [4]. The reviewer using the eCTAS smartphone application was an experienced paramedic (with 31 years of experience) who used CTAS in real time, provided CTAS education to paramedics, and was blinded to the documented CTAS levels on the call reports.

### 2.2. Participants/Subjects/Data

Data were collected from electronic Patient Care Records (ePCRs) that had a CTAS on contact and a CTAS on departure level assigned. The ePCRs were categorized into their respective CTAS on contact level, and a 95% confidence interval was calculated for each category to determine how many ePCRS would be reviewed. The ePCRs used in the study were identified by the assigned Ministry of Health call number and were selected using the = RAND (random selection tool) formula in Microsoft Excel, then sorted from the largest number to the smallest number. Once sorted, samples were selected in each category starting at the largest number until the sample size was met. Before running the random number generator, calls that had a CTAS on contact score of “0” were excluded. An assignment of “0” was specific to patients who were deemed to meet “obvious death” criteria, and the paramedic guidelines state that no CTAS level is to be assigned [1] or where a CTAS on contact or departure is not documented. Once this process was completed, the ePCR data were provided to the rater for assessment.

### 2.3. Measurement Methods and Procedure

**FIGURE 1:**
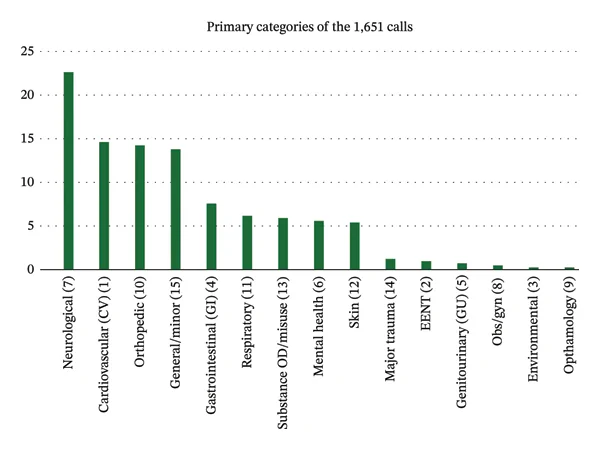
CTAS categories—Pacific Rim Nursing Consultants Inc.

A total of 192,326 records (Figure 1) were collected from ePCRs of the nine paramedic services under the RPPEO. The calls were categorized according to the CTAS on contact level, and the sample size was chosen to ensure a 95% CI. Based on the total number of call reports for each CTAS on contact level, the rater analyzed the following number of calls for each CTAS level category: CTAS 1—*n* = 300, CTAS 2—*n* = 352, CTAS 3—*n* = 350, CTAS 4—*n* = 345, and CTAS 5—*n* = 304. Exclusions that did impact the sample size, totaling *n* = 269 calls, were made for the following call types: interfacility transfers, discharge to home or hospice, end‐of‐shift crew relief during off‐load delay, and missing data. The missing data included ePCRs that had blank fields for primary problems, incident history, assessment, and/or vital signs, making a CTAS determination not possible for the independent rater. Tools used included Microsoft Excel and Minitab 21.4.2 (64 bit), VassarStats [17], and an electronic CTAS smartphone app. Using the primary problem designation on the ePCR, calls were placed into one of the 15 CTAS complaint groups listed on the eCTAS smartphone app. There are 17 complaint categories (Table 1) used by CTAS; however, the smartphone application combines the three ENT categories, making 15 categories [18].

**TABLE 1:** App categories and primary problem.

Categories and primary problem
Cardiovascular (CV)
ENTN
Environmental
Gastrointestinal (GI)
Genitourinary (GU)
Mental health
Neurological
Obs/Gyn
Ophthalmology
Orthopedic
Respiratory
Skin
Substance OD/misuse
Major trauma
General/minor

### 2.4. Data Analysis

Once collected in the Excel spreadsheet, the various primary problem description(s) were grouped into the corresponding CTAS complaint category and given a number from 1 to 15 [18] (Table 2).

**TABLE 2:** Cohen’s kappa and interpretation.

Cohen’s kappa	Interpretation
0	No agreement
0.10–0.20	Slight agreement
0.21–0.40	Fair agreement
0.41–0.60	Moderate agreement
0.61–0.80	Substantial agreement
0.81–0.99	Near‐perfect agreement
1	Perfect agreement

All calls that were further excluded were given a CTAS level of “0” for filtering purposes. Columns were also added to capture the change in CTAS between contact and departure, and the numeric difference of that change for both CTAS comparators. Columns were also added to capture the difference between CTAS and eCTAS for both contact and departure. Minitab 21.4.2 (64 bit) and VassarStats were used to run the data analysis for the quadratic‐weighted Cohen’s kappa (*k*) coefficient. The *p* value was calculated to determine the statistical significance of the results, with a *p* < 0.05 meaning that the results were not by random chance. The four columns, CTAS on contact, CTAS on departure, eCTAS on contact, and eCTAS on departure, were imported into the analysis software to run the analysis. Columns were also added to look at the number of calls by paramedic service, sex, the comparison of CTAS on contact compared to CTAS on departure, number of calls for each primary problem description, final primary problem description, and the breakdown of the smartphone application categories. Undertriage was evaluated for any call that was greater than one CTAS level below the appropriate CTAS level.

### 2.5. Ethics

Toronto Metropolitan University, Research Ethics Board (REB) assessed this study as a quality improvement project, and REB approval to use the data for secondary research purposes was granted by “*REDACTED”* following the review of the protocol. The REB #2023–494, Dated: December 22, 2023.

## 3. Results

The results returned by the data analysis software showed (Table 3) that for CTAS on contact compared to eCTAS on contact the overall quadratic‐weighted Cohen’s kappa (*k*) results were substantial at 0.78 (SE = 0.007, 95% CI: 0.7698–0.797) with a raw agreement of 55.24%, 95% CI (52.8 to 57.66) overall match between raters.

**TABLE 3:** Attribute agreement analysis for CTAS/eCTAS on contact.

Between appraisers assessment agreement					
# Inspected	# Matched	Percent		95% CI	
1651	912	55.24		(52.80, 57.66)	
# Matched: All appraisers’ assessments agree with each other					

**Kappa with quadratic weighting—contact—all**					

Observed kappa	Standard error		0.95 confidence interval		
			Lower limit	Upper limit	
0.7834	0.007		0.7698	0.797	
0.9585	Maximum possible quadratic‐weighted kappa, given the observed marginal frequencies				
0.8173	Observed as proportion of maximum possible				

**Proportions of agreement**				**0.95 CI of observed**	

**Category**	**Maximum possible**	**Chance expected**	**Observed**	**Lower limit**	**Upper limit**

1	0.9967	0.1001	0.8157	0.7688	0.8551
2	0.9034	0.1126	0.3929	0.3493	0.4383
3	0.6705	0.1453	0.2767	0.2438	0.3122
4	0.6696	0.0915	0.1779	0.1456	0.2154
5	0.9178	0.0966	0.436	0.3873	0.4858
Composite	0.8952	0.2016	0.5524	0.528	0.5765

The data show that 38 or 12% of CTAS 2 calls were documented as either CTAS 4 or 5 by paramedics, as opposed to 26 or 7% documented using the smartphone app. There was also a greater number of CTAS 4 and 5 calls documented by paramedics, 649 or 39%, versus 510 or 31% documented by the rater using the smartphone app. The quadratic‐weighted Cohen’s kappa (*k*) statistic showed near‐perfect agreement for CTAS 1 on contact between raters at (0.81), fair agreement for CTAS 2 (0.39) and CATS 3 (0.27), slight agreement for CTAS 4 (0.17), and moderate agreement for CTAS 5 (0.43) calls. The overall (*k*) score shows that there is substantial agreement between raters with a score of 0.78.

Likewise, for CTAS on departure compared to eCTAS on departure (Table 4), the overall quadratic‐weighted Cohen’s kappa (*k*) results were substantial at 0.76 (SE = 0.0081, 95% CI: 0.7534–0.785) with a raw agreement of 52.88%, 95% CI (50.44–55.31) match between raters.

**TABLE 4:** Attribute agreement analysis for CTAS/eCTAS on departure.

Between appraisers assessment agreement										
# Inspected					# Matched		Percent		95% CI	
1651					873		52.88		(50.44, 55.31)	
# Matched: All appraisers’ assessments agree with each other.										

**Kappa with quadratic weighting—contact—all**										

**Observed kappa**			**Standard error**				**0.95 confidence interval**			

							**Lower limit**		**Upper limit**	

0.7692			0.0081				0.7534		0.785	
0.9457	Maximum possible quadratic‐weighted kappa, given the observed marginal frequencies									
0.8134	Observed as proportion of maximum possible									

**Proportions of agreement**								**0.95 CI of observed**		

**Category**		**Maximum possible**		**Chance expected**		**Observed**		**Lower limit**		**Upper limit**

1		0.8985		0.0825		0.7119		0.656		0.7621
2		0.8711		0.12		0.3699		0.3286		0.4133
3		0.6909		0.1533		0.2807		0.2482		0.3156
4		0.6939		0.093		0.1785		0.1463		0.2158
5		0.8774		0.0989		0.4455		0.3971		0.495
Composite		0.8831		0.2051		0.5288		0.5044		0.5531

The data show that there were 36, or 11%, of CTAS 2 calls that were documented as either CTAS 4 or 5 by paramedics, as opposed to 27, or 7%, documented by the rater using the smartphone app. There was also a greater number of CTAS 4 and 5 calls documented by paramedics, 661 or 40%, versus 517 or 31% documented by the smartphone app. The quadratic‐weighted Cohen’s Kappa (*k*) statistic showed substantial agreement for CTAS 1 on contact between raters (0.71), fair agreement for CTAS 2 (0.36) and CTAS 3 (0.28), slight agreement for CTAS 4, and moderate agreement for CTAS 5 (0.44) calls. The overall quadratic‐weighted Cohen’s kappa (*k*) shows that there is substantial agreement between raters with a score of 0.76.

Each separate CTAS complaint category for both CTAS/eCTAS on contact and CTAS/eCTAS on departure was also analyzed. If we consider the gastrointestinal complaint category, as there was greater disagreement between the raters, the results showed a 42.4% match for CTAS/eCTAS on contact and a 44.0% match for CTAS/eCTAS on departure. Subsequently, the respective CTAS levels showed that CTAS 4 and 5 calls, when combined, totaled CTAS on contact of 52 or 42% versus eCTAS on contact of 26 or 21%. The CTAS/eCTAS on departure was again similar, with 51 or 41% and 27 or 22%, respectively. For the substance overdose/misuse complaint category, the CTAS/eCTAS on contact was in stark contrast to the CTAS/eCTAS on departure. There was a 60.2% match for calls on contact as opposed to a 47.9% match for calls on departure. In this category, the quadratic‐weighted Cohen’s kappa (*k*) results showed substantial agreement (0.72) overall for contact and only moderate agreement (0.54) for departure. There was near‐perfect agreement (0.82) for CTAS 1 on contact and fair agreement (0.30) for CTAS 1 on departure.

## 4. Discussion

To date, this is the first study to examine interrater agreement between paramedic documentation and a paramedic using a smartphone application in a Canadian context. The current investigation found that the most acute patients at CTAS level 1 for both CTAS/eCTAS on contact indicated near‐perfect agreement, and CTAS/eCTAS on departure indicated substantial agreement between raters. However, the degree of agreement for the other CTAS levels ranged more broadly, from fair to moderate agreement. These findings are consistent with other studies that utilized a similar design [6, 8, 12, 14]. Several factors likely contributed to the increased variability in CTAS scores for CTAS levels 2, 3, 4, and 5. For instance, data entries in the eCTAS application may vary depending on the user [4]. Howlett et al. refer to an inappropriate interpretation of the pain scale and missing vital signs as reasons for a lack of consistency between raters in these particular cases [14]. Similarly, Davis et al. found that removing pain symptoms from the CTAS algorithm skewed the distribution of CTAS scores toward lower acuity categories [19]. For instance, we found that the gastrointestinal category was relatively equal at 42.4% and 44.0% in the number of calls that matched for contact and departure, in contrast to the substance OD/misuse category, which was 60.2% on contact and 47.9% on departure. The discrepancy is likely because the use of a high‐risk substance, such as an opioid, is assigned as a CTAS level 2 by the app, even if everything else is within normal limits. In contrast, the Ontario Prehospital CTAS Paramedic Guide looks at the clinical signs and does not consider the substance as a modifier, resulting in disagreement with the CTAS level assignment. Studies have found that the CTAS modifiers make a significant difference to appropriate CTAS level assignment [3, 14] and appear to be the most significant contributor to the variability in CTAS scores [6]. Consistent with the paramedic CTAS guide, applying modifiers was associated with increased triage consistency, particularly for nonspecific complaints such as fever, pain, and general weakness when using computer or electronic decision support tools [8]. A few other studies have shown that when training was provided prior to implementation, there was higher agreement among raters [8, 9, 12]. Correspondingly, Dallaire et al. found that when they used experienced ED nurses without recent certification or recertification, they only received “moderate agreement” between nurses [20]. In this study, the finding that paramedics assigned lower acuity scores more frequently than the smartphone application warrants careful consideration. The documentation of 12% of CTAS 2 patients as CTAS 4 or 5 by paramedics, compared to only 7% by the app, exposes the subjective nature of the CTAS system and the potential for over or undertriage. This pattern extended across all acuity levels, with paramedics documenting nearly 40% of calls as CTAS 4 or 5 compared to 31% by the electronic application, validating subjectivity about consistency in triage assessment.

The need for ongoing training and education regarding patient complaints and how to effectively use CTAS tools may help to improve the assignment of CTAS levels and decrease the risk of undertriage.

### 4.1. Clinical Implications

Practitioners should look to use a CTAS guide when completing documentation as part of a continuous quality improvement initiative. While the nature and environment of a paramedic patient encounter will have the paramedic assigning CTAS by memory, committing to a guide when completing paperwork could reiterate the appropriate application of the CTAS modifiers. A unique aspect of this study is that each call type was categorized into the individual CTAS complaint categories and assigned a corresponding number. This allowed for an analysis of each category and the level of agreement generated. The near‐perfect agreement for CTAS level 1 patients is clinically significant, as it demonstrates consistent identification of the most critically ill patients who require immediate intervention. However, the variability in agreement for CTAS levels 2–5 may have implications for patient flow, resource allocation, and treatment prioritization, though clinical outcome studies are needed to determine whether these discrepancies translate to meaningful differences in patient care [6, 13].

The RPPEO should consider using these findings to generate training opportunities and examples for complaint categories that may indicate a gap in knowledge of either the complaint or the application of the CTAS levels. As such, CTAS level assignments should become a regular part of the quality review process that is conducted by the RPPEO and individual paramedic services.

### 4.2. Research Implications

Future studies should examine how paramedics assign CTAS levels in the field. These data could be collected through electronic surveys as part of the regular training cycle, which would improve efficiency in data collection. Further review of an eCTAS app designed for paramedic application should be explored. Having an app specific to the paramedic application of CTAS combined with the Medical Directive app could have multiple benefits. This could reduce the cost and improve availability, as it is currently only available through the Apple App Store, and it could reduce the workload burden of paramedics having to open multiple apps to review guides and medical directives.

### 4.3. Strengths and Limitations

This study had several strengths. This was the first study to examine interrater agreement between paramedic documentation and paramedics using a smartphone application. This is important as governments aim to improve healthcare effectiveness and efficiency through technological advancement. This study was conducted in Ontario, Canada, with a large sample size, thus improving the generalizability of the study implications. This study also identified gaps in CTAS assignments, therefore providing insight into areas for improvement in paramedic education. A major limitation of this study was the simplification of patients’ complaints, which may have influenced the accuracy of CTAS assignment. The CTAS categories are based on the patient’s chief complaint; however, the rater used the “Primary Problem Description and Final Problem Description” to pick which CTAS category to use. This decision simplified the data entry into the smartphone app as a complaint of “lift assist” or “MVC,” which does not translate into any CTAS complaint category, whereas the primary problem of musculoskeletal injury points the rater to the correct category. Another limitation was the exclusion of 269 calls from the analysis because of specific call types and incomplete data. The nature and extent of incomplete/missing data could not be fully characterized; therefore, no sensitivity analyses were performed to assess the potential bias associated with missing data in the results. Also, cases with incomplete information in the ePCRs were excluded, which reduced the sample size and may have affected the generalizability of the study findings. All cases were assessed by a single evaluator, which represents a study limitation. This approach may introduce evaluator‐dependent bias and limits our ability to assess interrater reliability. Future studies would benefit from multiple independent evaluators to enhance the robustness and reproducibility of findings.

## 5. Conclusion

There are limited studies on the use of CTAS in the field by paramedics. The results of this study demonstrate fair to moderate agreement between raters. Ambiguous complaint categories, such as gastrointestinal or mental health complaints, require more scrutiny when they are assessed. An understanding of how the CTAS modifiers involving pain, fever, and vital signs, as well as instances of high‐risk substance use, contribute to the variability in agreement is needed. Incorporating decision support tools should be encouraged, and improvements to the ePCR to flag disagreement in CTAS levels for review at the time of documentation will provide a feedback mechanism. Clarity in CTAS assignment by paramedics will help validate the MPDS quality review process. Alternate destination, PPS agreements, and nonconveyance protocols should use caution when CTAS scores are used as a factor to determine inclusion in these pathways. The results support the identification of potential areas for future quality improvement and training evaluation, but do not independently establish training deficiencies or patient safety consequences.

## Funding

There was no funding.

## Conflicts of Interest

The authors declare no conflicts of interest.

## Supporting Information

Additional supporting information can be found online in the Supporting Information section.

## Supporting information


**Supporting Information** STROBE‐checklist‐v4‐combined June 23 2026.doc.

## Data Availability

The data that support the findings of this study are available on request from the corresponding author. The data are not publicly available due to privacy or ethical restrictions.
