# Supplementary material for: Interrater Agreement Between Paramedic CTAS Scores and an Electronic CTAS Tool: A Retrospective Study Across Eastern Ontario
Source: Emerg Med Int. 2026 Sep 7;2026:3209833. doi: 10.1155/emmi/3209833 (PMC13550991; doi:10.1155/emmi/3209833)
Supplement: Supplementary file 1 [file EMMI-2026-3209833-s001.docx]

# Supplementary Table 1. STROBE Statement completed checklist (from von Elm *et al.* ^1^).

| **SECTION/TOPIC** | **ITEM No.** | **STROBE Recommendation** | **OUR PAPER** |
| --- | --- | --- | --- |
| **Title and**  **abstract** | 1 | (a) Indicate the study’s design with a  commonly used term in the title or the abstract | (a) See title and abstract on page 1 |
|  |  | (b) Provide in the abstract an  informative and balanced summary of what was done and what was found | (b) See abstract on page 1 |
| **Introduction** |  |  |  |
| Background / rationale | 2 | Explain the scientific background and rationale for the investigation being reported | See Introduction pages 1-5 |
| Objectives | 3 | State specific objectives, including any prespecified hypotheses | See last paragraph of Introduction on page 5 |
| **Methods** |  |  |  |
| Study design | 4 | Present key elements of study design early in the paper | See Study design on page 6 |
| Setting | 5 | Describe the setting, locations, and relevant dates, including periods of recruitment, exposure, follow-up, and data collection | See Study design on page 6 |
| Participants | 6 | (a) Give the eligibility criteria, and the sources and methods of selection of participants. Describe methods of follow-up | See Study design, Participants/Subjects/Data  on page 6 |
|  |  | (b) For matched studies, give matching criteria and number of exposed and unexposed | (b) Not applicable |
| Variables | 7 | Clearly define all outcomes, exposures, predictors, potential confounders, and effect modifiers. Give diagnostic criteria, if applicable | See Participants/ Subjects/Data, Measurement methods and procedure  on pages 6-7 |
| Data sources / measurement | 8 | For each variable of interest, give sources of data and details of methods of assessment (measurement). Describe comparability of assessment methods if there is more than one group | See Participants/ Subjects/Data, Measurement methods and procedure  on pages 6-7 |
| Bias | 9 | Describe any efforts to address potential sources of bias | Not applicable  See Measurement methods and procedure and Data analysis  on pages 7-8 and page 12 as limitation |
| Study size | 10 | Explain how the study size was arrived at | See Study design, Participants/Subjects/Data  on page 6-7, paragraph 1 of ‘Results’ section |

| Quantitative variables | 11 | Explain how quantitative variables were handled in the analyses. If applicable, describe which groupings were chosen and why | See Measurement methods and procedure and Data analysis  on pages 7-8 |
| --- | --- | --- | --- |
| Statistical methods | 12 | (a) Describe all statistical methods, including those used to control for confounding | See Measurement methods and procedure and Data analysis  on pages 7-8 |
|  |  | (b) Describe any methods used to  examine subgroups and interactions | See Measurement methods and procedure and Data analysis  on pages 7-8 |
|  |  | (c) Explain how missing data were  addressed | See Study design, Participants/Subjects/Data  on page 6 and page 12 (as limitation) |
|  |  | (d) If applicable, explain how loss to  follow-up was addressed | (d) Not applicable |
|  |  | (e) Describe any sensitivity analyses | (e) Not applicable |
| **Results** |  |  |  |
| Participants | 13 | (a) Report numbers of individuals at each stage of study— e.g. numbers potentially eligible, examined for eligibility, confirmed eligible, included in the study, completing follow-up, and analysed | (a) Please Section 2.3 measurement methods and procedure on page 7. Also, Results section on page 8 |
|  |  | (b) Give reasons for non-participation at each stage | (b) Not applicable |
|  |  | (c) Consider use of flow diagram | (c) Figure 1 |
| Descriptive data | 14 | (a) Give characteristics of study participants (e.g. demographic, clinical, social) and information on exposures and potential confounders | (a) Paragraphs 1 page 8; and Table 3 and 4 |
|  |  | (b) Indicate number of participants with missing data for each variable of interest | See 4.3 Strengths and Limitations section on page 12 |
|  |  | (c) Summarise follow-up time (e.g. average and total amount) | (c) Not applicable |
| Outcome data | 15 | Report numbers of outcome events or summary measures over time | See Resultls section on pages 8-9 |
| Main results | 16 | (a) Give unadjusted estimates and, if applicable, confounder-adjusted estimates and their precision (e.g. 95% confidence interval). Make clear which confounders were adjusted for and why they were included | See Resultls section on pages 8-9 |
|  |  | (b) Report category boundaries when continuous variables were categorised | (b) Not applicable |

|  |  | (c) If relevant, consider translating estimates of relative risk into absolute risk for a meaningful time period | (c) Not applicable |
| --- | --- | --- | --- |
| Other analyses | 17 | Report other analyses done e.g. analyses of subgroups and interactions, and | Not applicable |
| **Discussion** |  |  |  |
| Key results | 18 | Summarise key results with reference to study objectives | See Discussion section 1^st^ few sentences of the section on page 9 |
| Limitations | 19 | Discuss limitations of the study, taking into account sources of potential bias or imprecision. Discuss both direction and magnitude of any potential bias | See 4.3 Strengths and Limitations section on page 12 |
| Interpretation | 20 | Give a cautious overall interpretation of results considering objectives, limitations, multiplicity of analyses, results from similar studies, and other relevant evidence | ‘Conclusion’ |
| Generalisability | 21 | Discuss the generalisability (external validity) of the study results | See 4.3 Strengths and Limitations section on page 12 |
| **Other** |  |  |  |
| Funding | 22 | Give the source of funding and the role of the funders for the present study and, if applicable, for the original study on which the present article is based | ‘Funding’ section |

**Reference**

1) von EE, Altman DG, Egger M, Pocock SJ, Gotzsche PC, Vandenbroucke JP. The Strengthening the Reporting of Observational Studies in Epidemiology (STROBE) statement: guidelines for reporting observational studies. *Lancet* 2007; **370**(9596): 1453-1457
